# Analysis of Fertility Preservation by Ovarian Tissue Cryopreservation in Pediatric Children in China

**DOI:** 10.3389/fendo.2022.930786

**Published:** 2022-06-29

**Authors:** Xiangyan Ruan, Jiaojiao Cheng, Juan Du, Fengyu Jin, Muqing Gu, Yanglu Li, Rui Ju, Yurui Wu, Huanmin Wang, Wei Yang, Haiyan Cheng, Long Li, Wenpei Bai, Weimin Kong, Xin Yang, Shulan Lv, Yuejiao Wang, Yu Yang, Xin Xu, Lingling Jiang, Yanqiu Li, Alfred O. Mueck

**Affiliations:** ^1^ Department of Gynecological Endocrinology, Beijing Obstetrics and Gynecology Hospital, Capital Medical University, Beijing Maternal and Child Health Care Hospital, Beijing, China; ^2^ Department of Thoracic Surgery and Surgical Oncology, Children’s Hospital, Capital Institute of Pediatrics, Beijing, China; ^3^ Department of Surgical Oncology, Beijing Children’s Hospital, Capital Medical University, National Center for Children’s Health, Beijing, China; ^4^ Department of Pediatric Surgery, Children’s Hospital, Capital Institute of Pediatrics, Beijing, China; ^5^ Department of Obstetrics and Gynecology, Beijing Shijitan Hospital, Capital Medical University, Beijing, China; ^6^ Department of Gynecological Oncology, Beijing Obstetrics and Gynecology Hospital, Capital Medical University, Beijing Maternal and Child Health Care Hospital, Beijing, China; ^7^ Department of Obstetrics and Gynecology, Peking University People’s Hospital, Beijing, China; ^8^ Department of Gynecology and Obstetrics, First Affiliated Hospital of Xi’an Jiaotong University, Xi’an, China; ^9^ Department of Women’s Health, University of Tuebingen, University Women’s Hospital and Research Centre for Women’s Health, Tuebingen, Germany

**Keywords:** ovarian tissue cryopreservation, children, fertility preservation, ovarian tissue transplantation, reproduction, endocrine function, gonadal toxicity

## Abstract

**Background:**

Ovarian tissue cryopreservation (OTC) is the only method of fertility preservation (FP) in prepubertal girls, but the experience remains limited. This study investigates the effectiveness and feasibility of FP of OTC in children facing gonadotoxicity treatment in Chinese first ovarian tissue cryobank.

**Procedure:**

OTC and evaluation of 49 children ≤14 years old in the cryobank of Beijing Obstetrics and Gynecology Hospital, Capital Medical University, from July 2017 to May 19, 2022, were analyzed retrospectively. We compared children’s general characteristics, follicle numbers, and hormone levels with and without chemotherapy before OTC.

**Results:**

The age of 49 children at the time of OTC was 7.55 (1–14) years old. There were 23 cases of hematological non-malignant diseases, eight cases of hematological malignant diseases, four cases of gynecological malignant tumors, one case of neurological malignant tumors, one case of bladder cancer, five cases of sarcoma, three cases of mucopolysaccharidosis, one case of metachromatic leukodystrophy, two cases of dermatomyositis, one case of Turner’s syndrome. The median follicular count per 2-mm biopsy was 705. Age and AMH were not correlated (r = 0.084, *P =* 0.585). Age and follicle count per 2-mm biopsy was not correlated (r = −0.128, *P =* 0.403). Log10 (follicle count per 2-mm biopsy) and Log10 (AMH) were not correlated (r = −0.118, *P =* 0.456). Chemotherapy before OTC decreased AMH levels but had no significant effect on the number of follicles per 2-mm biopsy.

**Conclusions:**

OTC is the only method to preserve the fertility of prepubertal girls, and it is safe and effective. Chemotherapy before OTC is not a contraindication to OTC.

## 1 Introduction

The overall incidence of cancer in children has increased slightly (0.7% per year), and the reason is unclear (1). Because of significant treatment advances in recent decades, 85% of children with cancer now survive more than 5 years (1). Childhood cancer treatment may include surgery, chemotherapy, radiotherapy, and/or hematopoietic stem cell transplantation (HSCT) (2). Except for non-pelvic surgery, these treatments impair ovarian function, which is related to the oocyte/follicle DNA double-strand break (DSB) and apoptosis (3). Gonadal toxicity of anticancer therapy depends on the type, dose, and extent of chemotherapeutic agents and radiotherapy (4). HSCT is a standard treatment for hematological diseases, including high-dose chemotherapy with or without whole-body radiotherapy. Patients cured by myeloablative HSCT have a very high risk of premature ovarian insufficiency (POI) (5).

Decline or loss of fertility and POI are well-known side effects of anticancer therapy, and infertility is a significant concern for childhood cancer survivors (6). Estrogen deficiency also affects uterine development and increases the risk of osteoporosis, cardiovascular disease, and impaired cognitive function. In adolescent girls, POI also leads to developmental impairment and delayed puberty and affects self-esteem (7). Although fertility preservation (FP) in patients with cancer has become an essential issue in the clinic, previous studies have shown that young patients with cancer are not always adequately counseled about the potential adverse effects of cancer treatment on reproductive function and FP options nor are they referred to a fertility specialist (8). This issue has yet to be addressed in a proper manner, especially in low-income settings (9).

The ovarian tissue cryopreservation (OTC) technique is the only FP method for prepubertal girls. Cortical tissue is obtained by laparoscopic minimally invasive surgery under general anesthesia (10, 11). The adolescent or young adults could have ovarian stimulation and oocytes retrieval once they have recovered and before they develop to POI. In 2019, the American Society for Reproductive Medicine (ASRM) claimed that the OTC technique is no longer an experimental technique but has become standard clinical FP technology (12). Because of the small ovary size in children, the unilateral ovary is usually retrieved for OTC (13).

More than 200 babies have been born through OTC technology worldwide (14), and cryopreserved ovarian tissue from children has been successfully used to induce puberty (15). Recently, there have been reports of successful pregnancy after retransplantation of cryopreserved ovarian tissue at the age of 13 (16) and 9 (17) and in patients with acute lymphoblastic leukemia at the age of 14 (18). The International Guideline Harmonization Group pointed out that all children with cancer and their families have the right to be informed of the risk of gonadal damage and recommends that children and young patients who will receive a cumulative dose of 6,000~8,000 mg/m^2^ or greater alkylating agent, ovarian radiotherapy, and HSCT undergo FP of OTC (8).

The global clinical practice information on OTC of prepubertal girls and adolescent women is still limited, and the FP experience in children is limited compared to adults. To better apply OTC for prepubertal girls, the valuable experience of each center is worth reporting. This study mainly analyzed the age, disease, transport and cryopreservation, follicle number, and hormone level of 49 children who underwent OTC in the cryobank of Beijing Obstetrics and Gynecology Hospital, Capital Medical University, the first and largest OTC center in China. It compares the age, disease, transport and cryopreservation, follicle count, and hormone level of patients with or without chemotherapy before OTC.

## 2 Methods

### 2.1 Ethics Statement

The Ethics Committee approved OTC of Beijing Obstetrics and Gynecology Hospital, Capital Medical University (ethics code: 2017-KY-020-01; date: March 15, 2017) to provide centralized OTC and use up to 10% of ovarian tissue for quality control measures and patient-related research. Ovarian tissue was collected from clinical sub-centers and transferred to the ovarian tissue cryobank.

### 2.2 Retrieval, Transportation, and Preparation of Ovarian Tissue

**Figure 1:**
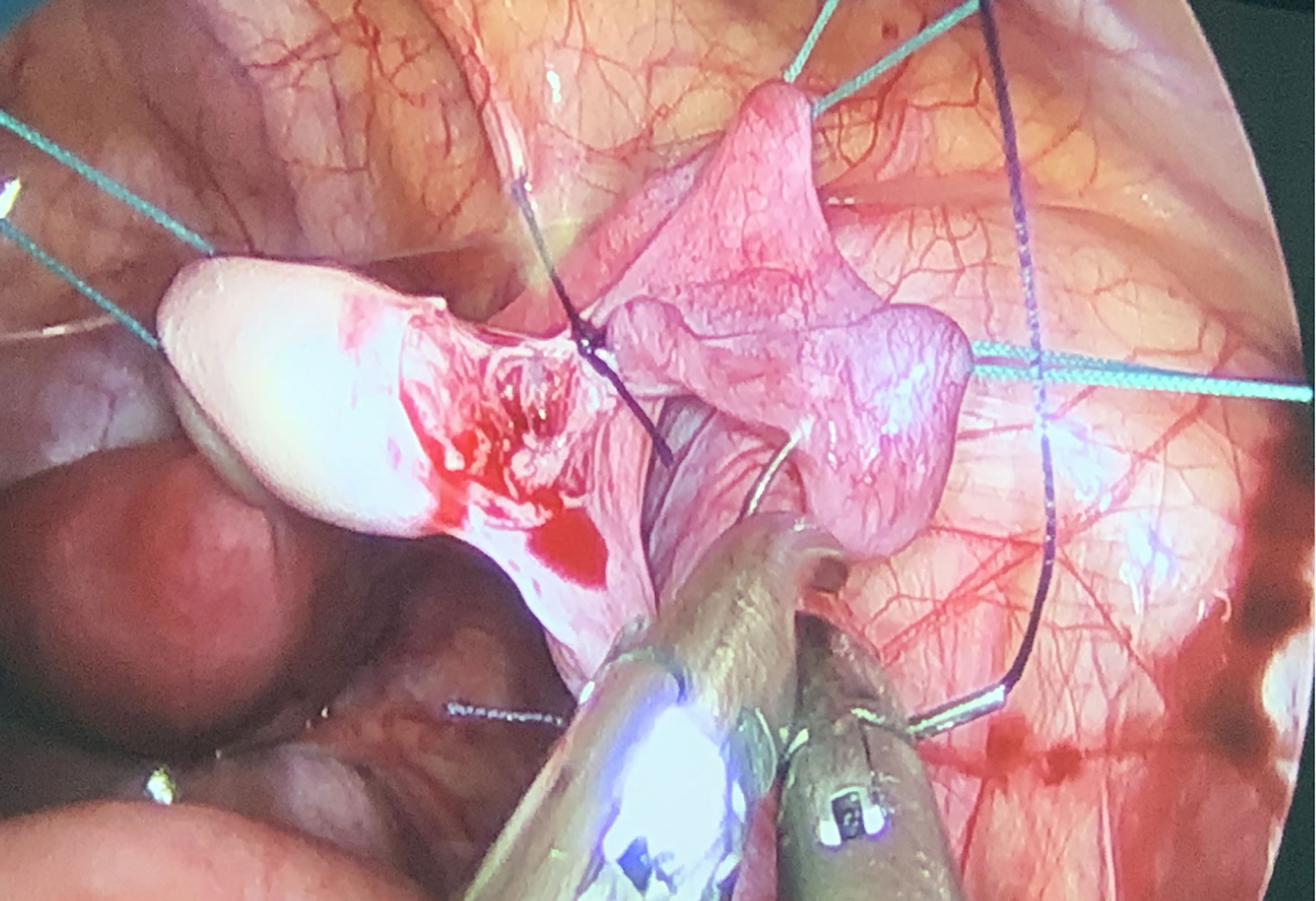
Intraoperative photo demonstrating ovarian anatomy in a 11-year-old female with chronic active Epstein-Barr virus infection.

Forty-one children who underwent OTC in the cryobank of Beijing Obstetrics and Gynecology Hospital, Capital Medical University, from July 2017 to May 19, 2022 (mean ± SD, range: 7.55 ± 3.64 years, 1–14 years) were selected as subjects. Twenty-four of them underwent a few cycles of chemotherapy before OTC to alleviate the symptoms of the disease and most of them reach the remission stage and plan to undergo HSCT. Because of the small size of the ovary in children (13), the amount of ovarian tissue retrieval is generally the unilateral ovary, equivalent to 50% of all ovarian tissue, *via* laparoscopy (**Figure 1**) or laparotomy (primary tumor resection at the same time).

The ovarian tissue was transferred to the cooled Custodiol immediately after retrieval. During ovarian tissue transport, the temperature was maintained at 4°C–8°C. The mean temperature reached the cryobank was 5.47°C, and the average transport time was less than 12 h. In a pollution-free environment, ovarian tissue was prepared in a sterile laminar flow cabinet at 4°C. The cortex was prepared to 1 mm thick, then cut into cortical slices of size about 6 mm × 3 mm, and cryopreserved for future transplantation. For the remaining cortical tissue, standardized cortical samples (diameter of 2 mm) were obtained from different areas using punches (PFM Medical AG, Cologne, Germany) for follicle density analysis and routine viability assay. After slow programmed freezing, the ovarian cortex was stored in a gas phase liquid nitrogen tank. The operation is according to the previously published protocol (19, 20).

### 2.3 Hormone Level Analysis Before Ovarian Tissue Cryopreservation

The levels of follicle-stimulating hormone (FSH), luteinizing hormone (LH), and estradiol (E2) in serum before OTC were determined by Centaur automatic chemiluminescence immunoassay produced by Bayer Company in the United States. AMH was determined using the AMH kit (Guangzhou Kangrun Company, China) by enzyme-linked immunosorbent assay. The intra-assay and inter-assay errors were 3.3% and 6.7%, respectively.

### 2.4 Analysis of Follicle Density

The count of surviving primordial and primary follicles was analyzed in standardized biopsied cortices, with circular cortical slices 2 mm in diameter collected from different cortex regions with a volume of 3.14 mm^3^ per biopsy. The follicles count per 2-mm biopsy, and the follicles density per mm^3^ were statistically analyzed. The follicular count assessment method is the same as the previous articles published by our team (21).

### 2.5 Statistical Analyses

SPSS 22.0 (IBM SPSS Statistics, IBM software) was used for data analysis. The data in accordance with normal distribution were expressed by “mean ± standard deviation”, the mean between groups was compared by independent sample t-test, and the data in accordance with non-normal distribution were represented by “median, range”. The Mann–Whitney U-test compared the median between groups. Spearman correlation analysis was used to analyze the variables that did not conform to the normal distribution. *P* < 0.05 indicates that the difference is statistically significant.

## 3 Results

### 3.1 Patient Characteristics

#### 3.1.1 Ages and Diagnosis

**Figure 2:**
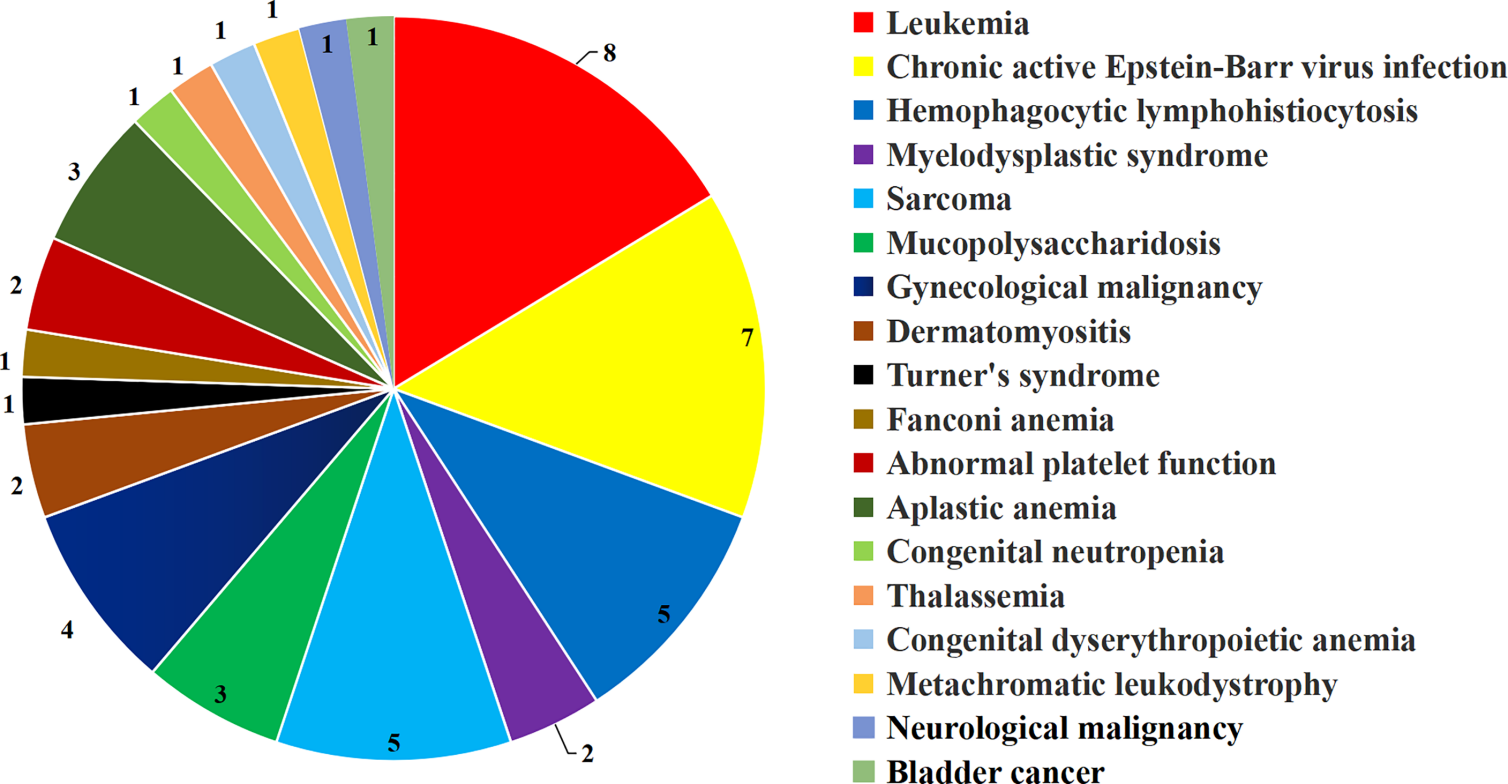
Disease classification of 49 children undergone OTC. OTC, ovarian tissue cryopreservation.

Characteristics of children with OTC are shown in **Table 1**. The age of 49 children was 7.55 ± 3.64 years old, range: 1 to 14 years. The disease distribution of 49 patients was (**Figure 2**): 23 cases of hematological non-malignant diseases, eight cases of hematological malignant diseases, four cases of gynecological malignant tumors, one case of neurological malignant tumor, one case of bladder cancer, three cases of mucopolysaccharidosis, one case of metachromatic leukodystrophy, two cases of dermatomyositis, one case of Turner’s syndrome, and five cases of sarcoma. Thirty-six patients (36 of 49, 73.5%) with hematological non-malignant diseases, hematological malignant diseases, mucopolysaccharidosis, and dermatomyositis were cryopreserved because of planned HSCT. The number of patients with or without chemotherapy before OTC was 24 and 25, respectively. The cycles of chemotherapy before OTC were 3 (1–11) (median, range).

**Table 1:** Patient characteristics and comparison with chemotherapy and without chemotherapy before OTC.

Characteristics	Overall children	Chemo before OTC	No-Chemo before OTC	*P*-value
Age (mean ± SD) (n)	7.55 ± 3.64 (49)	8.54 ± 4.06 (24)	6.60 ± 2.94 (25)	0.061
Transport temperature (mean ± SD) (n)	5.47 ± 1.34 (49)	5.76 ± 1.53 (24)	5.20 ± 1.10 (25)	0.156
The proportion of ovarian retrieval in the total ovary (median, range) (n)	0.50, 0.15-0.75 (48)	0.50, 0.25-0.75 (24)	0.50, 0.15-0.50 (24)	0.957
Number of cryopreserved cortex pieces (mean ± SD) (n)	20.52 ± 7.63 (48)	21.42 ± 8.04 (24)	19.63 ± 7.26 (24)	0.422
Follicle number per 2-mm biopsy (median, range) (n)	705, 122–3628 (45)	868, 158–2,250 (21)	507, 122–3628 (24)	0.290
Follicle density per mm^3^ (median, range) (n)	224.52, 38.85–1,155.41 (45)	276.43, 50.32–716.56 (21)	161.47, 38.85–1155.41 (24)	0.290
FSH (IU/L) before OTC (median, range) (n)	2.34, 0.00–17.66 (41)	2.20, 0.58–6.83 (21)	3.18, 0–17.66 (20)	0.182
LH (IU/L) before OTC (median, range) (n)	0.00, 0.00–63.23 (41)	0.10, 0.00–63.23 (21)	0.00, 0.00–4.26 (20)	0.270
E2 (pg/ml) before OTC (median, range) (n)	12.21, 11.80–326.72 (41)	12.28, 11.80–326.52 (21)	12.06, 11.80–93.13 (20)	0.834
AMH (ng/ml) before OTC (median, range) (n)	0.89, 0.06–5.94 (45)	0.27, 0.06–3.21 (23)	1.51, 0.4–5.94 (22)	0.000***

***refers to P < 0.001.

OTC, ovarian tissue cryopreservation; SD, standard deviation; FSH, follicle stimulating hormone; LH, luteinizing hormone; E2, estradiol; AMH, anti-Müllerian hormone.

#### 3.1.2 Number of Children Undergoing OTC Per Year

**Figure 3:**
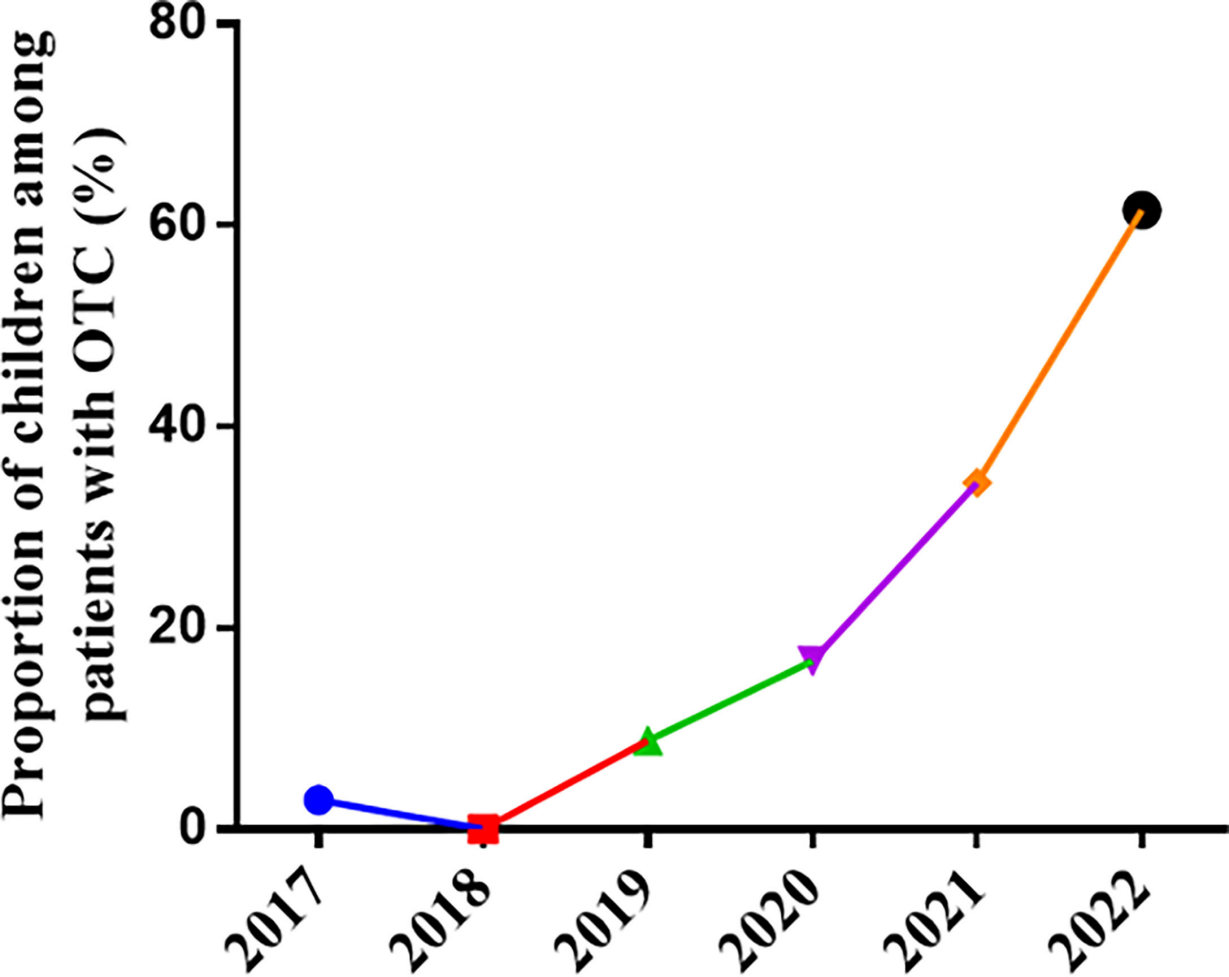
The proportion of children among patients with OTC per year. OTC, ovarian tissue cryopreservation.

From July 2017 to May 19, 2022, 52 children come to counseling OTC at our center, and 49 children have performed the OTC. Among the patients who underwent OTC in 2017, there was only one child patient (1 of 35, 2.9%), and none of the patients experienced OTC in 2018 (0). In 2019, there were five child patients (5 of 57 8.8%). In 2020, there were five child patients (6 of 36, 16.7%). In 2021, there were 21 children (21 of 61, 34.4%) and eight patients who underwent OTC in 2022 (16 of 26, 61.5%). The proportion of children in patients with cryopreserved ovaries increased significantly (**Figure 3**).

#### 3.1.3 Ovarian Tissue Retrieval, Transportation, Cryopreservation, and Follicle Density

In **Table 1**, the temperature of ovarian tissue transport to a centralized cryobank is 5.47 ± 1.34°C. The proportion of ovarian retrieval in the total ovary is 0.50, 0.15–0.75 (median, range), the number of cryopreserved cortex pieces is 20.52 ± 7.63 (mean ± SD), and the follicle number per 2-mm biopsy is 705, 122–3,628 (median, range). Follicle density per mm^3^ is 224.52, 38.85–1,155.41 (median, range).

#### 3.1.4 Hormone Levels Before OTC

In **Table 1**, FSH level before OTC is 2.34, 0.00–17.66 IU/L (median, range); LH level before OTC is 0.00, 0.00–63.23 IU/L (median, range); E2 level before OTC is 12.21, 11.80–326.72 pg/ml (median, range). The patients with the highest values of LH and E2 were in the same patient aged 14 years, with menarche at 11 years old. AMH level before OTC is 0.89, 0.06–5.94ng/ml (median, range).

#### 3.1.5 Correlation Analysis and Linear Regression Analysis

**Figure 4:**
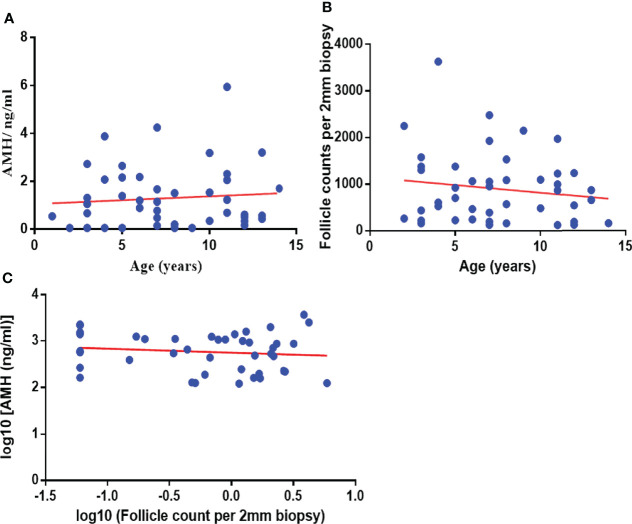
Correlation analysis and linear regression analysis between Age and AMH **(A)** (n = 45, r = 0.084, *P =* 0.585), between age and follicle count per 2-mm biopsy **(B)** (n = 45, r = −0.128, *P =* 0.403), between log10 (follicle count per 2-mm biopsy) and log10 (AMH) **(C)** (n = 45, r = −0.118, *P =* 0.456). AMH, anti-Müllerian hormone.

Age and AMH were not significantly correlated (n = 45, r = 0.084 P *=* 0.585). Age and follicle count per 2-mm biopsy were not significantly correlated (n = 45, r = −0.128, *P =* 0.403). Log10 (follicle count per 2-mm biopsy) and Log10 (AMH) were not significantly correlated (n = 45, r = −0.118, *P =* 0.456) (**Figure 4**).

### 3.2 Comparison of Chemotherapy and No Chemotherapy Before OTC

#### 3.2.1 Ages and Diagnosis

**Figure 5:**
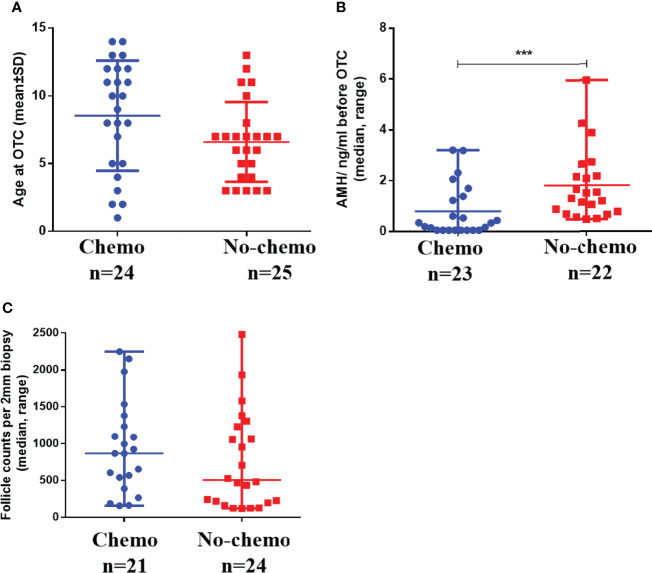
Comparison of age **(A)**, AMH **(B)**, and follicle count per 2-mm biopsy **(C)** in children with and without chemotherapy before OTC. *** refers to P<0.001. OTC, ovarian tissue cryopreservation.

In **Table 1**, There was no significant difference in the age of patients with or without chemotherapy before OTC (8.54 ± 4.06, 6.60 ± 2.94, *P =* 0.061) (**Figure 5**). The main diseases in patients with chemotherapy (n = 24) before OTC were eight cases of hematological non-malignant diseases, seven cases of hematological malignant diseases (leukemia), five cases of sarcoma (one case of Ewing’s sarcoma and four cases of rhabdomyosarcoma), one case of gynecological malignant tumor, one case of neurological malignant disease, and one case of bladder cancer. Malignant diseases accounted for 62.5% (15 of 24). The main diseases of patients without chemotherapy (n = 25) before OTC were 15 hematological non-malignant diseases, three cases of gynecological malignant tumors, six other non-malignant diseases, and one hematological malignant disease. Malignant diseases accounted for 16% (4 of 25).

#### 3.2.2 Ovarian Tissue Retrieval, Transportation, Cryopreservation, and Follicle Density

In **Table 1**, there was no significant difference in transport temperature (mean ± SD, 5.76 ± 1.53 vs. 5.20 ± 1.10), the proportion of ovarian retrieval in the total ovary (median, range, 0.50, 0.25–0.75 vs. 0.50, 0.15–0.50), the number of cryopreserved cortex pieces (mean ± SD, 21.42 ± 8.04 vs. 19.63 ± 7.26), follicle number per 2-mm biopsy (median, range, 868, 158–2250 vs. 507, 122–3628), and follicle density per mm^3^ (median, range, 276.43, 50.32–716.56 vs. 161.47, 38.85–1,155.41) between the two groups with or without chemotherapy before OTC (all *P* > 0.05) (**Figure 5**).

#### 3.2.3 Hormone Levels Before OTC

In **Table 1**, There was no significant difference in FSH, LH, and E2 levels between the two groups with or without chemotherapy before OTC (all *P*>0.05). AMH levels in patients with chemotherapy before OTC were significantly lower than those without chemotherapy (median, range: 0.27, 0.06–3.21 vs. 1.51, 0.48–5.94, *P =* 0.000) (**Figure 5**).

## 4 Discussion

Our center is the first ovarian tissue cryobank in China and is also currently the largest ovarian tissue cryobank in China. More than 400 cases of ovarian tissue have been successfully cryopreserved, 10 cases of adult ovarian tissue have been successfully transplanted, and the ovarian function has been restored after transplantation (19). One of the adult patients with MDS has successfully conceived naturally and delivered a healthy baby girl through OTC and transplantation. This is also the first baby born in China through OTC and transplantation (22). With the cooperation of pediatrics, the proportion of OTC of children in our center has increased over the past 2 years.

Cyclophosphamide and other alkylating agents are commonly used to treat cancer in children. They induce apoptosis of cancer cells by destroying DNA and inhibiting cell metabolism, DNA replication, and transcription but cause vascular toxicity to ovaries and direct DNA damage to growing and dormant follicles, resulting in acute ovarian failure (23). Radiotherapy also increases the risk of infertility, depending on age, ovarian reserve, total radiation dose, and radiation plan. Head radiation destroys the hypothalamus and pituitary function, leading to hypogonadism. Fifty percent of follicular loss can be caused by direct pelvic radiation of 2 Gy, and pelvic irradiation can lead to myometrial fibrosis. More than 25-Gy radiation seems to lead to irreversible damage to the uterus (24).

HSCT is a standard treatment option that usually cures severe benign and malignant diseases. Of the 49 children who underwent OTC for FP in this study, 73.5% planned HSCT treatment. In this study, seven children with chronic active EB virus infection underwent OTC. As far as we know, there are only two cases of OTC in patients with this disease (25). This study describes for the first time that OTC was performed in children with mucopolysaccharidosis and included different types, such as type I, type IVa, and type IH. We also performed OTC for five patients with hemophagocytic lymphohistiocytosis. It was reported that the ovaries of three patients with hemophagocytic lymphohistiocytosis were cryopreserved, but two patients died before the application of cryopreserved ovarian tissue (26). The study has reported that the ovarian tissue of adult patients with hemophagocytic lymphohistiocytosis was cryopreserved, ovarian function recovered after OTT, and the pregnancy was successful (27). The HSCT of leukemia patients is applied in remission, and the patients may have received chemotherapy for months or years before HSCT (28). Referral to fertility counseling before HSCT is the most important for patients. The study has shown that, even after receiving HSCT in childhood, fertility counseling and assessment of residual fertility potential can provide opportunities for FP (28).

AMH is produced almost entirely by granulosa cells of small antral follicles between 5 and 8 mm, reflecting gonadotropin-independent follicle genesis (29). In adults with regular menstrual cycles, serum FSH can be considered a marker of pituitary function. The peak level of serum AMH is in puberty or early adulthood. It has been proved that AMH correlates with the antral follicle count in healthy women. Still, it is not recommended as the primary monitoring method to evaluate POI in the childhood cancer group (30, 31). The continuous longitudinal follow-up study is significant (32). The hypothalamus–pituitary–ovary axis is stationary in childhood, and the evaluation of FSH, LH, E2, and AMH levels is not practical in children. There was no significant correlation between age and AMH level and the number of follicles in this study. There was no significant correlation between AMH level and the number of follicles.

ASRM stressed the importance of surgical techniques in retrieving ovarian tissue and the importance of tissue preparation for cryopreservation, which is the core of the quality of cryopreservation and ultimately crucial to the success of the future application of ovarian tissue to restore fertility (33). Laparoscopic ovarian tissue retrieval has been proven safe for children and adults, with low intraoperative and postoperative risks. Because of the small size of the ovaries in children, laparoscopic unilateral ovariectomy is the first choice, maximizing the number of cortical tissue for cryopreservation and future retransplantation and avoiding bleeding complications. Multiple cortical slices can allow multiple attempts at OTT, and if the hormone recovery effect is weakened, then OTT can be repeated (34). Ovarian tissue retrieval for OTC does not seem to affect patients significantly. Unilateral ovariectomy may advance the age of menopause by about 1.8 years, which may be due to a compensatory mechanism, leading to a low recruitment rate of primordial follicles (25, 35).

At present, the low temperature (4°C to 8°C) is a widely used standard tissue transportation method. Low temperatures decrease cellular metabolism in ovarian tissue, reduce cellular oxygen demand and consumption, and preserve tissue without vascular perfusion (36, 37). Because of the high density of follicles in children, we generally prepared ovarian cortical slices with the size of 6 mm × 3 mm × 1 mm, which is consistent with the international community (38). At present, ovarian tissue slow-programming cryopreservation is internationally recognized as the gold standard procedure for OTC (38). Except for the five reported babies born after vitrification of ovarian tissue, other more than 200 new births were born through slow-programming of OTC (39).

For patients with a high risk of ovarian tissue transplantation, such as leukemia and non-Hodgkin’s lymphoma, part of the ovarian tissue was taken for histopathology to evaluate whether there was cancer cell infiltration before OTC. In addition, the thawed part of the cryopreserved ovarian tissue before transplantation was analyzed by histological and molecular biology techniques to evaluate that there were no cancer cells in the cryopreserved ovarian tissue (40). No evidence of malignant cell contamination was observed in cryopreserved ovarian tissues from patients with non-metastatic solid tumors and Hodgkin’s lymphoma (41, 42). The evidence cited in the existing guidelines does not indicate cancer recurrence in transplanted ovarian tissue (40, 43). There were no pregnancy complications or congenital abnormalities in women after transplantation of cryopreserved ovarian tissue (44). However, the safety of ovarian tissue at high risk of the disease still needs to be thoroughly evaluated.

During the preparation of ovarian tissue, the small antral follicles in the medulla are destroyed, and cumulus-oocyte complexes (COCs) are released into the medium. These COCs can be recovered and matured *in vitro* to obtain MII oocytes and cryopreserved for future application (2, 45). Live birth from the source of the oocytes has been reported (46, 47). Most *in vitro* maturation (IVM) results are from adults, and more research is needed to determine how to mature preadolescent gametes into high-quality eggs. Telfer et al. have grown and matured human primordial follicles to the preantral and antral follicle stages (48). More work is required before *in vitro* culture can be clinically applied and offered. In the future, artificial ovaries will reduce the possibility of reintroducing malignant cells into the body and eliminate the need for ovarian tissue autotransplantation, such as *in vitro* growth and IVM of primordial follicles (49).

Most of the patients who underwent chemotherapy before OTC were malignant diseases, and the proportion of malignant diseases was higher than that of patients without chemotherapy before OTC. This is consistent with the results of another study, which showed that 95% of patients who receive chemotherapy before OTC have hematological malignant diseases (50). There was no significant difference in the number of follicles, FSH, LH, and E2 between children undergoing chemotherapy before OTC and those without chemotherapy, but the level of AMH in children undergoing chemotherapy before OTC was significantly lower than that in children without chemotherapy. AMH is mainly secreted by granulosa cells of small antral follicles, and chemotherapy may significantly damage metabolically active follicles, such as growing follicles. Therefore, the level of AMH in patients with chemotherapy before OTC is significantly lower than that in patients without chemotherapy, and it may take 1~3 years to recover (31). For diseases with a high risk of cancer cell contamination in the ovary, such as leukemia, OTC after complete remission with chemotherapy reduces the risk. In our study, the population is heterogeneous, the sample number is limited, the cryopreserved ovarian tissue in childhood has not been transplanted, and we cannot prove that chemotherapy before OTC does not affect the outcome of OTT. However, other studies have shown that chemotherapy before OTC has little effect on the number of follicles, does not affect the outcome of OTT, and is no longer a contraindication for OTC (50, 51).

Still, the study has shown that the interaction between immature ovarian tissue and the hypothalamus and pituitary is similar to that of the adult ovary, indicating that ovarian maturation needs appropriate FSH and LH stimulation. These results support the use of OTC in prepubertal girls. The current study does not cover the functional lifespan of cryopreserved prepubertal ovary grafts after transplantation. Long-term follow-up is still needed to monitor whether a large number of non-growing follicles transplanted due to younger age will prolong the grafted life (52).

## 5 Conclusion

Advances in clinical oncology care in children have greatly improved survival and now pose challenges to the long-term quality of life during survival, including fertility and hormonal function. Pediatric surgeons need to continue to advocate FP, incorporate FP methods before and early treatment, and perform ovarian tissue surgery on children at high risk of POI if safe and necessary. OTC in children seems to be a safe procedure and needs to be confirmed in large prospective studies to provide data for developing guidelines for OTC in children.

## Data Availability Statement

The raw data supporting the conclusions of this article will be made available by the authors, without undue reservation.

## Ethics Statement

The studies involving human participants were reviewed and approved by Beijing Obstetrics and Gynecology Hospital, Capital Medical University. Written informed consent to participate in this study was provided by the participants’ legal guardian/next of kin.

## Author Contributions

All authors qualify for authorship by contributing substantially to this article. XR: project leader, project supervision, interpretation of results, provided critical comments, and revised the first draft. JC: article preparation, ovarian tissue transportation, preparation, and cryopreservation. JD, FJ, MG, and YLL: ovarian tissue preparation and cryopreservation. RJ, YRW, HW, HC, WY, LL, WB, WK, XY, and SL: biopsied ovarian tissue. YW, YY, XX, LJ, YQL: ovarian tissue transportation, AM: experimental supervision, interpretation of results, and article revision. All authors contributed to the article and approved the submitted version.

## Funding

This study was supported by Beijing Natural Science Foundation (7202047), Capital’s Funds for Health Improvement and Research (2020-2-2112), Beijing Municipal Administration of Hospitals’ Ascent Plan (DFL20181401), Capital’s Funds for Health Improvement and Research (2016-2-2113), Beijing Municipal Science and Technology Commission (Z161100000516143), and Beijing Municipal Administration of Hospitals Clinical Medicine Development of Special Funding Support (XMLX201710).

## Conflict of Interest

The authors declare that the research was conducted in the absence of any commercial or financial relationships that could be construed as a potential conflict of interest.

## Publisher’s Note

All claims expressed in this article are solely those of the authors and do not necessarily represent those of their affiliated organizations, or those of the publisher, the editors and the reviewers. Any product that may be evaluated in this article, or claim that may be made by its manufacturer, is not guaranteed or endorsed by the publisher.
